# Appraising community driven health research with Aboriginal and Torres Strait Islander communities: a scoping review using the Aboriginal and Torres Strait Islander Quality Appraisal Tool

**DOI:** 10.1093/heapro/daac077

**Published:** 2022-09-26

**Authors:** Brett Biles, Jessica Biles, Kate Friere, Linda Deravin, Jayne Lawrence, Aryati Yashadhana

**Affiliations:** Faculty of Medicine and Health, University of New South Wales, Sydney, NSW, Australia; Charles Sturt University, School of Nursing, Paramedicine and Healthcare Sciences, Albury, NSW, Australia; Three Rivers UDRH, Charles Sturt University, Faculty of Science and Health, Albury, NSW, Australia; Charles Sturt University, School of Nursing, Paramedicine and Healthcare Sciences, Albury, NSW, Australia; Charles Sturt University, School of Nursing, Paramedicine and Healthcare Sciences, Albury, NSW, Australia; Centre for Health Equity Training Research & Evaluation, UNSW, Sydney, NSW, Australia; Centre for Primary Health Care & Equity, UNSW Medicine & Health, Sydney, NSW, Australia; School of Social Sciences UNSW, Sydney, NSW, Australia; Ingham Institute for Applied Medical Research, Liverpool, NSW, Australia

**Keywords:** community driven, health research, Aboriginal and Torres Strait Islander communities

## Abstract

Most research involving Aboriginal and Torres Strait Islander peoples has been conducted by non-Indigenous people and has not been a positive experience for many Aboriginal and Torres Strait Islander communities. This scoping review maps approaches to health research involving Aboriginal and Torres Strait Islander peoples and communities in Australia from the last two decades. A literature search found 198 papers, of which 34 studies met the inclusion criteria. The Aboriginal and Torres Strait Islander Quality Appraisal Tool was then used to map the quality of the reported community driven research. The Quality Appraisal Tool privileges, Aboriginal and Torres Strait Islander people’s epistemologies and ethical research governance. The findings reported on strengths and identified areas for improvement in reporting community driven research.

## INTRODUCTION

The Declaration on the Rights of Indigenous people’s evidences that peoples have the right to ‘*maintain, control, protect and develop their cultural heritag*e’ (United Nations, 2007). This is applicable and relevant to all aspects of life, including research. Contemporary approaches to research related to Aboriginal and Torres Strait Islander peoples are supported through national policy directives and involve established and documented agreements that clearly outline expectations from Aboriginal and Torres Strait Islander peoples, communities and research teams. National ethical governance protocols suggest that Aboriginal and Torres Strait Islander peoples and communities should lead and drive research projects (NHMRC, 2018b). Aboriginal and Torres Strait Islander peoples have a long history of being the world’s most researched people (Smith, 2013).

Research governance is facilitated through six core values that should be reflected in all aspects of any research project. Responsibility, reciprocity, respect, spirit and integrity, equity and cultural continuity are values that underpin all Aboriginal and Torres Strait Islander research (NHMRC, 2018b). These six core values seek to provide researchers with an understanding of how to maintain and build relationships, ensuring justice and fairness and no discriminatory practice. All groups and people within the research relationship should have equal power where net benefits including respect and responsibility are evidenced with the community where the research is being undertaken (NHMRC, 2018a). Through this research process, the spirit and integrity of Aboriginal and Torres Strait Islander peoples are upheld. Researchers in the developmental phase of the research framework need to ensure that these reportable values are threaded throughout the project (NHMRC, 2018a). Ethical reporting mandates researchers must provide a detailed description of how research is controlled and driven by community needs (NHMRC, 2018b).

In 2020, the Australian Institute of Aboriginal and Torres Strait Islander Studies (AIATSIS) updated its ethical guidelines. AIATSIS originally published ethical guidelines in 1999, based on a new approach to situate Aboriginal and Torres Strait Islander people as partners in research. In 2012, there was a further update, which included 14 guiding principles for ethical research with Aboriginal and Torres Strait Islander people. The most recent iteration of these guidelines has been structured in a framework to reflect the expected standards of researching with, and not on, Aboriginal and Torres Strait Islander people. While governance frameworks mandate and frame Aboriginal and Torres Strait Islander research, it is not clear how the espoused frameworks in peer reviewed health research are enacted. Therefore, the basis of this scoping review is to examine the approaches for community driven health research in Aboriginal and Torres Strait Islander communities used during the past two decades.

### Design

A scoping review was chosen to ensure a comprehensive mapping of community driven health research with Aboriginal and Torres Strait Islander communities. Scoping reviews identify the available knowledge in a specific area and therefore highlight key terms and gaps in knowledge (Peters *et al.*, 2015).

## METHODS

The Preferred Reporting Items for Systematic Reviews and Meta Analyses (PRISMA) (Moher *et al.*, 2009) and Aboriginal and Torres Strait Islander Quality Appraisal Tool (QAT) (Harfield *et al.*, 2020) guided and framed the approach.

### Keywords and search strategies

For this scoping review, extensive time was spent discussing and defining terms. Two terms that were considered relevant to health research with Aboriginal and Torres Strait Islander communities and important to the team were *community control* and *community driven*. Community controlled is defined through National Aboriginal Community Controlled Health (NACCHO) (National Aboriginal Community Controlled Health (NACCHO), 2021) as:

‘… a process which allows the local Aboriginal community to be involved in its affairs in accordance with whatever protocols or procedures are determined by the Community.’

The following statement sheds light on the concept of community driven from a research perspective:

‘The research commenced with firmly set values about how the research would be conducted as a community-directed approach to disability research, but without a pre-determined framework or hard-wired set of methods.’ (Avery, 2020)

These two definitions guided the scoping review and keyword search. Keywords and phrases were searched through CINAHL, MEDLINE (OVID), SCOPUS and Informit Health Collection. With the support of a university librarian, search terms were tested and assessed for validity, ensuring that replication was possible and resulting in 198 eligible papers for review. Journals were shared with the research team via an online Endnote library.

### Study selection and paper extraction

Four team members attended to the title/abstract review seeking peer reviewed, full text articles that were focussed on Aboriginal and Torres Strait Islander peoples. A 20-year date range was appropriate to align with the Close the Gap policy. Papers were grouped into three categories: ‘relevant’, ‘not relevant’ and ‘not sure’. To ensure rigour, the papers deemed ‘not relevant’ and ‘not sure’ were discussed in a wider team meeting, before exclusion, with congruence in decision-making paramount. Collaborative yarning as a method framed the researcher meetings, ensuring that different perspectives were acknowledged in the articles review. Exploring different ideas and understanding of each article was considered vital during the paper extraction phase of the research (Bessarab, 1996). Decisions were noted in a PRISMA diagram. Eligible papers were then screened using the Aboriginal and Torres Strait Islander Quality Appraisal Tool (QAT) (Harfield *et al.*, 2020).

### Aboriginal and Torres Strait Islander Quality Appraisal Tool

The QAT is a validated tool used to guide research focussed on Aboriginal and Torres Strait Islander peoples from the perspective of Aboriginal and Torres Strait Islander communities (Harfield *et al.*, 2020). The tool privileges Aboriginal and Torres Strait Islander people’s epistemologies and ethical research governance (Harfield *et al.*, 2020), aligning with the overall focus of this scoping review. The tool consists of 14 questions as cited in Table 1.

**Table 1:** Summary of Harfield *et al.* (Harfield *et al.*, 2020)

Question (N)	The Aboriginal and Torres Strait Islander Quality Appraisal Tool Questions
1	Did the research respond to a need or priority determined by the community?
2	Was community consultation and engagement appropriately inclusive?
3	Did the research have Aboriginal and Torres Strait Islander research leadership?
4	Did the research have Aboriginal and Torres Strait Islander governance?
5	Were local community protocols respected and followed?
6	Did the researchers negotiate agreements in regard to rights of access to Aboriginal and Torres Strait Islander peoples’ existing intellectual and cultural property?
7	Did the researchers negotiate agreements to protect Aboriginal and Torres Strait Islander peoples’ ownership of intellectual and cultural property created through the research?
8	Did Aboriginal and Torres Strait Islander peoples and communities have control over the collection and management of research materials?
9	Was the research guided by an Indigenous research paradigm?
10	Does the research take a strength-based approach, acknowledging and moving beyond practices that have harmed Aboriginal and Torres Strait peoples in the past?
11	Did the researchers plan and translate the findings into sustainable changes in policy and/or practice?
12	Did the research benefit the participants and Aboriginal and Torres Strait Islander communities?
13	Did the research demonstrate capacity strengthening for Aboriginal and Torres Strait Islander individuals?
14	Did everyone involved in the research have opportunities to learn from each other?

The Aboriginal and Torres Strait Islander Quality Appraisal Tool Questions.

At the recommendation of Harfield’s user guide (Harfield *et al.*, 2020), a pilot of the tool was undertaken on one paper. All team members participated in this process and associated yarning session to validate the researchers’ understanding of the 14 criteria points. From this, the team reviewed each paper twice using the tool’s criterion scale of ‘Yes’, ‘Partial’, ‘No’ and ‘Unclear’ (Harfield *et al.*, 2020). The results were combined where two categories of either partial/yes or unsure/no papers were allocated. Any discrepancies between the two initial reviewers were resolved by a third team member.

## RESULTS

**Fig. 1:**
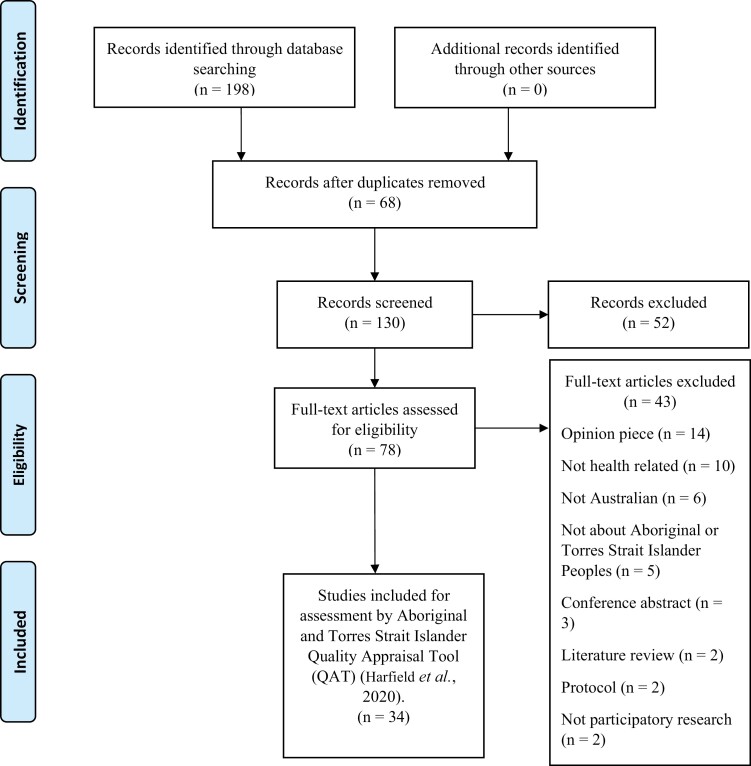
PRISMA flow diagram.

A total of 198 papers were found through the database searches (Figure 1). Once duplicates were removed and the initial title and abstract screening completed, 78 full text papers were retrieved. A further 44 papers were excluded following a full text review. Thus, 34 studies were found to have met the inclusion criteria and included in the review.

Figure 1 depicts a summary of the search strategy results in a PRISMA diagram (Moher *et al.*, 2009). Table 1 provides a summary of the questions in the Aboriginal and Torres Strait Islander Quality Appraisal Tool (Harfields et al., 2020) and characteristics described in Table 2. Time of publication spanned between 2003 and 2020 with a mixture of qualitative and quantitative papers. Fifty per cent (17/34) of the papers used participatory action research (PAR) or a hybrid of PAR and 14% (5/34) used mixed methods studies. Overall, 52% of the studies (18/34) cited an Aboriginal Human Research Ethics approval within the body of the paper.

**Table 2:** Table of characteristics

Author	Year	Identified methodology	Aboriginal HREC approval number identified	Number of participants (*N* =)
Couzos *et al.*	2015	Community-based participatory research	Identified	2522
Dudgeon *et al.*	2017	Participatory action research	Identified	457
Scrine *et al.*	2020	Participatory action research	Identified	138
Turner *et al.*	2019	A multi-case comparative study design (quantitative and qualitative data)	Identified	132
Weetra *et al.*	2019	Mixed methods	Identified	344
Hedges *et al.*	2020	Observational study	Identified	1011
Peake *et al.*	2020	Participatory action research	Not identified	Unclear
Chambers *et al.*	2018	Qualitative summative evaluation	Identified	32
Haynes *et al.*	2019	Participatory action research	Unable to determine	Unclear
McCalman *et al.*	2009	Participatory action research	Not identified	Interviews 6, focus groups 31, surveys 26
Sherwood and Kendall	2013	Community collaborative participatory action research with Indigenous conceptual framework	Identified	Unclear
Miller *et al.*	2015	Participatory action research	Identified	Unclear
Mooney-Somers *et al.*	2012	Community-based participatory research	Not identified	45
Adams *et al.*	2012	Participatory action research	Not identified	10
Fehring *et al.*	2019	Qualitative and quantitative methods	Not identified	97
Gauld *et al.*	2011	Participatory action research	Not identified	65
Dimitropoulos *et al.*	2020	Quantitative design	Identified	88
Munns *et al.*	2017	Participatory action research	Identified	14
Tyrrell *et al.*	2003	Mixed methods	Not identified	Unclear
Munro *et al.*	2017	Post-intervention evaluation	Not identified	53
Nasir *et al.*	2017	Community-based participatory research	Not identified	Unclear
Champion *et al.*	2008	Participatory action research	Not identified	30
McDonald *et al.*	2014	Participatory systems	Identified	Unclear
Mooney-Somers *et al.*	2009	Qualitative	Identified	45
Josif *et al.*	2012	Participatory action research	Not identified	Unclear
Reeve *et al.*	2015	Mixed methods	Identified	Unclear
Fuller *et al.*	2012	Participatory action research case studies	Identified	42
Panaretto *et al.*	2006	Quantitative design	Not identified	196
Sushames *et al.*	2017	Mixed methods	Not identified	12
Sayers *et al.*	2016	Evaluation study qualitative methods	Not identified	20
Passmore *et al.*	2017	Mixed methods	Identified	122
Graham and Clough	2019	Intervention and evaluation study through qualitative methods	Not identified	407
Hefler *et al.*	2019	Grounded action approach	Identified	13
Irving *et al.*	2017	Survey design	Identified	49

Note: Studies listed by total Harfield score in alphabetical order.

Table 3 shows the results of screening using the QAT. The average score of the 34 studies was 8/14. Five studies (15%) achieved a full score on the appraisal tool and three studies (9%) were found not to have met any of the criteria (0/14).

**Table 3:** Screening of studies with the Aboriginal and Torres Strait Islander Quality Appraisal Tool (QAT)

First author and date	Aboriginal and Torres Strait Islander Quality Appraisal Tool Question number^a^														
	1.	2.	3.	4.	5.	6.	7.	8.	9.	10.	11.	12.	13.	14.	Total score
Couzos 2015	1	1	1	1	1	1	1	1	1	1	1	1	1	1	14
Dudgeon 2017	1	1	1	1	1	1	1	1	1	1	1	1	1	1	14
Scrine 2020	1	1	1	1	1	1	1	1	1	1	1	1	1	1	14
Turner 2019	1	1	1	1	1	1	1	1	1	1	1	1	1	1	14
Weetra 2019	1	1	1	1	1	1	1	1	1	1	1	1	1	1	14
Hedges 2020	1	1	1	1	1	0	1	1	1	1	1	1	1	1	13
Peake 2020	1	1	1	1	1	0	1	1	1	1	1	1	1	1	13
Chambers 2018	1	1	1	1	1	0	0	1	1	1	1	1	1	1	12
Haynes 2019	1	1	1	1	1	0	0	1	1	1	1	1	1	1	12
McCalman 2009	1	1	1	1	1	0	0	1	1	1	1	1	1	1	12
Sherwood 2013	1	1	1	1	1	0	1	1	1	1	1	1	1	0	12
Miller 2015	0	1	1	1	1	0	1	1	0	1	1	1	1	1	11
Mooney-Somers 2012	1	1	1	1	1	0	0	1	0	1	1	1	1	1	11
Adams 2012	1	1	1	1	0	0	0	0	1	1	1	1	1	1	10
Fehring 2019	0	1	0	1	1	1	1	1	0	0	1	1	1	1	10
Gauld 2011	1	1	1	1	1	0	0	0	0	1	1	1	1	1	10
Dimitropoulos 2020	1	1	0	1	1	0	0	1	0	0	1	1	1	1	9
Munns 2017	0	1	1	1	0	0	0	0	1	1	1	1	1	1	9
Tyrell 2003	1	1	1	1	1	0	0	0	0	0	1	1	1	1	9
Munro 2017	1	1	1	0	1	0	0	0	0	1	1	1	1	0	8
Nasir 2017	1	1	0	1	1	0	0	1	0	1	1	1	0	0	8
Champion 2008	0	1	1	0	1	1	0	1	1	0	0	0	0	1	7
McDonald 2017	0	1	0	1	0	0	0	0	0	1	1	1	1	1	7
Mooney-Somers, Mahers 2009b	0	1	1	0	0	0	0	0	0	1	1	1	1	1	7
Josif 2012	1	1	0	1	0	0	0	0	0	1	1	1	0	0	6
Reeve 2015	1	1	0	1	1	0	0	0	0	1	1	0	0	0	6
Fuller 2012	0	0	0	1	1	0	0	0	0	0	0	1	1	1	5
Panaretto 2006	1	0	0	0	0	0	0	0	0	0	1	1	0	0	3
Sushames 2017	0	0	0	0	1	0	0	0	0	1	1	0	0	0	3
Sayers 2016	0	0	0	0	0	0	0	0	0	0	1	1	0	0	2
Passmore 2017	0	0	0	0	0	0	0	0	0	0	0	1	0	0	1
Graham 2019	0	0	0	0	0	0	0	0	0	0	0	0	0	0	0
Hefler 2019	0	0	0	0	0	0	0	0	0	0	0	0	0	0	0
Irving 2017	0	0	0	0	0	0	0	0	0	0	0	0	0	0	0

Harfield *et al.* (Harfield *et al.*, 2018).

Note: Studies listed by quality score in alphabetical order.

Refer to Table 1 for details of the question number

**Fig. 2:**
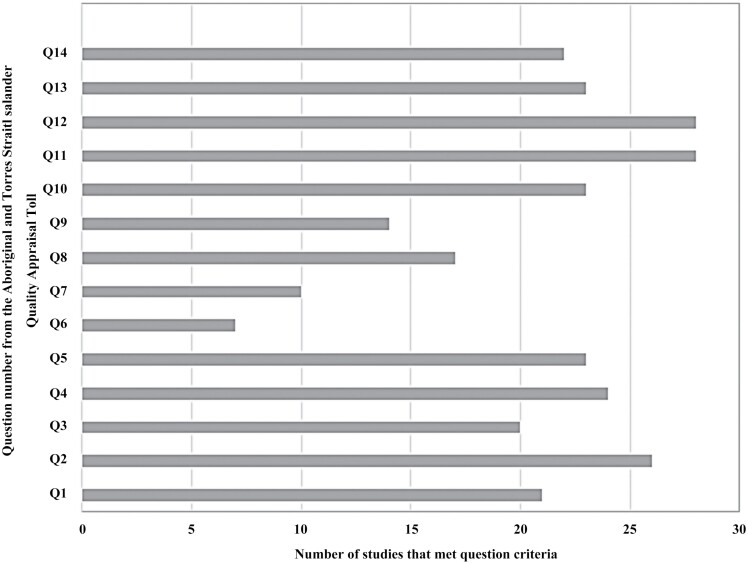
Total score per criteria from The Aboriginal and Torres Strait Islander Quality Appraisal Tool (QAT).

Figure 2 shows an analysis based on the 14 criteria of the quality appraisal tool. In summary, over 75% of studies met or partially met the following three criteria: translation of findings into sustainable changes (28/34, 82%) (criteria 11), benefit the participants and Aboriginal and Torres Strait Islander communities (82%) (criteria 12) and inclusive community consultation and engagement (76%) (criteria 2). In contrast, four criteria were met or partially met by 50% or less of the studies. Less than 25% of studies (7/34, 21%) met the criteria addressing existing intellectual and cultural property (criteria 6). Criteria 7 (created intellectual and cultural property) was met or partially met by 10/34 (29%); criteria 9 (use of an Indigenous research paradigm) was met or partially met by 14/34 (41%); and criteria 8 (First Nation control over collection and management of research materials) was met or partially met by 17/34 (50%).

## DISCUSSION

This study sought to comprehensively appraise published community driven health research with Aboriginal and Torres Strait Islander communities in the past two decades. Importantly, the AIATSIS Code of Ethics for Aboriginal and Torres Strait Islander Research was updated in 2020 with the goal of promoting ethical and responsible research practices, increasing the contribution of Aboriginal ways of knowing, being and doing, and ensuring that research does not harm Aboriginal and Torres Strait Islander people (AIATSIS, 2020). The AIATSIS code is structured under four key principles with each having a set of clearly defined responsibilities for conducting Aboriginal and Torres Strait Islander research. The validated QAT is foundational in assessing quality from the perspective of Aboriginal and Torres Strait Islander peoples (Harfield *et al.*, 2020). The AIATSIS ethical guidelines clearly state that self-determination and Aboriginal and Torres Strait Islander leadership are minimum standards that are required for researching with Aboriginal and Torres Strait Islander peoples. Despite these guidelines being in place since 1999, much health-related research still fails to partner with or have any significant input from Aboriginal and Torres Strait Islander people (Human Rights and Equal Opportunity Commission, 2007; Sherwood, 2010). Therefore, the discussion will focus on key strengths and areas for improvement that will guide researchers reporting on research focussed on Aboriginal and Torres Strait Islander peoples and communities.

### Community consultation and engagement

According to Harfield *et al*. (Harfield *et al*., 2019), research should be tailored to meet the needs of communities, with a focus on community consultation and involvement. Specifically, the papers that scored well in Harfield’s guide (total score >13), also achieved positive scores in community consultation and engagement and importantly provided context for methods that supported community consultation and engagement. There was a direct correlation between Harfield’s performance (Couzos *et al.*, 2015; Dudgeon *et al.*, 2017; Turner *et al.*, 2019; Weetra *et al.*, 2019; Scrine *et al.*, 2020) and community consultation and engagement. In papers that scored higher results, initial proceedings were prefaced by outlining what consultative communication looked like and who was involved in creating the research (Couzos *et al.*, 2015; Dudgeon *et al.*, 2017; Turner *et al.*, 2019; Weetra *et al.*, 2019; Scrine *et al.*, 2020).

Community consultation was often described as seeking ‘*local*’ [(Dudgeon *et al.*, 2017), p. 134] support and input during the foundational stages of the research design and being led by Aboriginal researchers and/or an Aboriginal steering group (Weetra *et al.*, 2019; Scrine *et al.*, 2020). Aligning with the NHMRC (NHMRC, 2018b) values, consultation often extended to local co-researchers or partner organizations (Dudgeon *et al.*, 2017; Turner *et al.*, 2019; Peake *et al.*, 2020; Scrine *et al.*, 2020). The papers described co-researchers as having local community cultural knowledge that supported all phases of the research process (Dudgeon *et al.*, 2017; Scrine *et al.*, 2020), providing an emerging theoretical framework of community consultation and engagement.

Consultation required the ability to nurture environments where mutual trust, safety and reciprocal learning could be fostered (Turner *et al.*, 2019; Hedges *et al.*, 2020). How this aligns with and complements national research funding guidelines remains in question. However, modes of communication that supported consultation included social, collaborative and research yarning. These were seen to increase engagement and consultation within the community (Peake *et al.*, 2020).

Regular and ongoing communication between co-researchers was imperative to consultation and engagement (Peake *et al.*, 2020). This extended to communication between the community and co-researchers. Importantly, communication needed to extend beyond the research to become meaningful. For example, sharing information about family and friends was important in developing an environment of respect and trust. This is important in establishing rapport and trust with Aboriginal communities.

Interestingly, community consultation and engagement were often supported by western models of research, such as PAR (Couzos *et al.*, 2015; Dudgeon *et al.*, 2017; Peake *et al.*, 2020; Scrine *et al.*, 2020) with few studies citing decolonizing Indigenous methodology underpinning the study (Sherwood and Kendall, 2013). Despite the NHMRC (NHMRC, 2018b) governing principles, very few formal research agreements were incorporated into the reportable findings of papers (Couzos *et al.*, 2015; Hedges *et al.*, 2020). This is not to say that they did not occur, but they were not acknowledged in the traditional research reporting mode, that is, journal publications. Research agreements were described as legally binding agreements that define the interests of the involved parties and associated relationships. These agreements were complemented by formal ethics approvals.

### Research leadership

The role of leadership in consultation and engagement is important. Weetra *et al.* (Weetra *et al.*, 2019) discuss how Aboriginal and Torres Strait Islander researchers play a key role in facilitating opportunities for ­consultation and engagement. Research leadership that enabled engagement, led to higher rates of recruitment, which built confidence for the research team.

Aboriginal and Torres Strait Islander peoples’ steering committees were an important component of research leadership. Through the PAR methodology, steering committees were described as guiding decision-making through the research process (Weetra *et al.*, 2019; Hedges *et al.*, 2020) and were an important demonstration of research leadership.

Hedges *et al.* (Hedges *et al.*, 2020) reported on the number of Aboriginal and Torres Strait Islander people vs. non-Indigenous people involved in the research project. Dudgeon *et al.* (Dudgeon *et al.*, 2017) summarized that community ownership of the research and community consultation enable the opportunity for Aboriginal and Torres Strait Islander people’s leadership and control in the research process to align with the NHMRC (NHMRC, 2018a) principles.

### Sustainable changes and research benefits

As one of the most researched population groups (Smith, 2013), Aboriginal and Torres Strait Islander people have been subject to over examination and review by researchers seeking to gain an understanding of this group. Historically, this has resulted in research being done on, rather than with, Aboriginal and Torres Strait Islander peoples. An encouraging aspect of this review was that a high proportion of articles (*n* = 21) identified that the need for the research was in fact a community identified need or priority (Harfield *et al.*, 2020). If driven by community need then it would be assumed participation and engagement by the community would be forthcoming. Most of the articles where community need was seen as a priority identified that the research resulted in mutual learning from the researchers, the community and the population group, which in turn was seen as a positive effect in making sustainable changes in either policy or practice. Sherwood and Kendall, 2013; Chambers *et al.*, 2018; Peake *et al.*, 2020.

The Closing the Gap policies intent to improve the health outcomes of Aboriginal and Torres Strait Islander peoples was an open admission from the government that healthcare policy and practice had not been making a difference to health outcomes of Aboriginal and Torres Strait Islander people (Australian Government, 2009). Harfield *et al.* (Harfield *et al.*, 2020) indicate that good quality Indigenous-based research should encompass a knowledge transition plan and the results of any research should result in either policy or practice change. Of the articles reviewed, 28 claimed to have impacted either policy and/or practice. Embedding change in policy because of the research seeks to support Aboriginal and Torres Strait Islander people to influence future policy and have a voice in how healthcare services are provided.

Harfield *et al.* (Harfield *et al.*, 2020) indicate that an important component of good quality research undertaken in partnership with Aboriginal and Torres Strait Islander communities is that it should have an overall benefit to the community in which the research is being conducted. The research included in this review had a strong correlation with this criterion—28 articles provided evidence of research benefits to the community. Where research benefit was identified by the researchers for Indigenous communities, there was also evidence of the development of meaningful and ongoing partnerships (Sherwood and Kendall, 2013; Chambers *et al.*, 2018).

### Negotiated agreements to the rights of accessing existing intellectual and cultural property

According to Harfield *et al.* (Harfield *et al.*, 2020), all research projects should have a formal agreement that has been negotiated with Aboriginal and Torres Strait Islander peoples. The agreement is required to articulate the rights of access to existing intellectual and cultural property and data ownership. A large majority of the papers (*n* = 27) failed to meet this criterion. Fundamental to self-determination, is to respect, protect and maintain Indigenous people’s knowledge systems, practices, science innovations and cultural expressions and is done through identifying the rights of intellectual and cultural property. Weetra *et al.* (Weetra *et al.*, 2019) clearly articulated that an Aboriginal advisory group was set up for this research, which involved community leaders with expertise in policy, government and Aboriginal Community Controlled Health Services (ACCHS). Their role was to guide consultation, interpretation of data, protocol development and data collection. Research by Couzos *et al.* (Couzos *et al.*, 2015) clearly articulated and provided extensive detail around the types of research agreement and could be a best practice example. Couzos *et al.* (Couzos *et al.*, 2015) also clearly stipulated that agreement should conform to NACCHO data protocols.

### Negotiated agreements to protect ownership of intellectual and cultural property created through the research

According to the NHMRC guidelines (NHMRC guidelines, 2018b), knowledge created through research is required to remain the intellectual property of Aboriginal and Torres Strait Islander research contributors and their contribution should be acknowledged in research outputs. Given that criteria 6 had only 7 papers that met or partially met the criterion, it was not a surprise that only 10 papers met or partially met criterion 7. The fourth component of the AIATSIS code of ethical research, ‘Impact and Value’, clearly articulates the importance of having a shared agreement about the benefit, impact and value of research, which also highlights the importance of reciprocity in relation to cultural expressions and intellectual property. Meaningful engagement and collaboration are key components of Aboriginal and Torres Strait Islander research and must be present throughout all stages of the research process. A key part of engagement and collaboration is to ensure that agreements are in place to protect ownership of the intellectual and cultural property that has been created through the research.

Scrine *et al.* (Scrine *et al.*, 2020) demonstrated this process by having nine Aboriginal Elders as co-researchers who determined the research activities, including data collection methods, analysis and translation. The Elders’ authority led to a decolonizing approach, which ensured that the power and voice were given back to Aboriginal people—this aligns with Aboriginal ways of knowing, being and doing. Couzos *et al.* (Couzos *et al.*, 2015) ensured that data analysis, interpretations and publications were agreed on by the research team or through the project reference group.

### Aboriginal control of research

The control of research spaces by non-Aboriginal researchers contributes to poor translation of research findings into social change or improvements to health systems and processes (Bainbridge *et al.*, 2015). The dominance of Western biomedical epistemologies has shaped the Aboriginal health research space as a site of struggle; whereby Aboriginal access and contribution to, and more so ownership of research knowledge, is key to its decolonization (Smith, 2013). We found that half of the papers included in the synthesis (*n* = 17) scored 0 for the appraisal section addressing whether Aboriginal peoples and communities had control over the collection and management of research materials. This finding highlights a lack of mechanisms that enable Aboriginal ‘control’ of research, including a clear definition of its praxis. For example, six of the studies that did not include Aboriginal control of research, did include Aboriginal ‘research leadership’ (Tyrell et al., 2003; Mooney-Somers et al., 2009a; Gauld *et al.*, 2011; Adams *et al.*, 2012; Munns *et al.*, 2017, Munro *et al.*, 2017), revealing complexity and confusion over what these terms mean theoretically as opposed to in practice.

Examples of varying forms of Aboriginal control of research within the reviewed papers included having research agreements in place that ensured Aboriginal organizations (e.g. ACCHSs) had ownership of unanalyzed data, enabling new research requests to require their endorsement and approval (Couzos *et al.*, 2015); co-developing a set of project principles that included ‘community ownership (Dudgeon *et al.*, 2017); and collaboration with Aboriginal Elders or community members as co-researchers who determined data collection, analysis and translation processes (Hedges *et al.*, 2020; Scrine *et al.*, 2020)’. A review of Aboriginal health research in Australia (Thomas *et al.*, 2014) concluded that the involvement and funding of Aboriginal people as researchers, improved ethical frameworks needed to enhance corresponding improvements in health outcomes.

Several papers that were scored as including an element of control, described processes that involved Aboriginal ‘guidance’ of the research process, via various forms of advisory or reference groups (Weetra *et al.*, 2019; Hedges *et al.*, 2020), or community forums (Chambers *et al.*, 2018). Clarifying the difference between ‘control’ and guidance is needed, as the former implies that there are mechanisms in place that enable Aboriginal organizations and communities to steer decision-making processes (including data collection and analysis), while the latter is less concrete and may or may not involve the incorporation of Aboriginal guidance or advice into decision-making processes (e.g. external researchers or research grant holders make the decisions).

Another element of ‘control’ includes ethical approval from an Aboriginal Human Research Ethics Committee (HREC), which 18 papers did not include. For example, one paper (Peake *et al.*, 2020) described involving Aboriginal people in research processes, and building authentic engagement using Aboriginal research paradigms yet did not obtain an Aboriginal HREC approval. Such approaches do not enable Aboriginal control from genesis, as gaining approval from a non-Aboriginal HREC to conduct Aboriginal research disempowers community control over the research process (Couzos *et al.*, 2015).

### Aboriginal research paradigms

Some papers (*n* = 18) used PAR approaches either in conjunction with, or to replace, Aboriginal research paradigms. Originally, PAR methodologies challenged positivist ideas of objective rigour, and sought to democratize knowledge (Brown and Tandon, 1983) through enabling participant involvement in, and shared access to research processes. PAR approaches may also involve seeking transformative change through social justice and activism (e.g. the ‘action’ in PAR); however, scholars have argued that researchers are often more interested in the R, leaving the A limited in its outcomes (Chatterton *et al.*, 2007). Regardless, PAR can align well with Aboriginal research paradigms, through centring lived experiences and voices of Aboriginal peoples and communities (for example, this was seen in Dudgeon *et al.*, 2017); however, they are not one and the same (Kendall *et al.*, 2011).

Of the papers included in this review, just under half (*n* = 15) were not guided by an Aboriginal research paradigm. Aboriginal research paradigms are varying and contextually dependent, yet similarities in ways of being, knowing and doing have been documented. Noonuccal woman Karen Martin-Booran Miraboopa (Martin-Booran Miraboopa, 2003) describes ways of being as existing within a network of relations among ‘entities’ that are reciprocal, through practices of relatedness and connectedness. They entail processes that allow expansion and contraction according to the social, political, historical and spatial dimensions of individuals, the group and interactions with outsiders [(Martin-Booran Miraboopa, 2003), p. 210]. Ways of doing show being and knowing in action, and therefore applying these approaches to research frames the way that data (knowledge) is obtained, analyzed and presented to wider audiences, connecting to aspects of ethics and control discussed previously.

Among the reviewed papers that did draw on an Aboriginal research paradigm, the inclusion of Aboriginal researchers and Elders in all or most phases of the research (including data analysis) was key. Approaches involved embedding community ways of knowing over research data (Couzos *et al.*, 2015), including Aboriginal interpretation and co-authorship of data (Hedges *et al.*, 2020) and the use of Aboriginal methodologies such as yarning (Chambers *et al.*, 2018; Peake *et al.*, 2020); respecting and centring the role of Aboriginal researchers including the obligation to maintain relationships beyond the study period (Weetra *et al.*, 2019); placing Aboriginal Elders at the centre of the research process ensuring alignment with culturally centred values and beliefs (Scrine *et al.*, 2020); and supporting linguistic sovereignty through the use of Aboriginal languages in preferred settings (Weetra *et al.*, 2019). Aboriginal research paradigms prioritize respect, cultural safety and the establishment of trusting working relationships, all of which take time. However, the temporality of research funding agreements can create barriers to the application of Aboriginal research approaches, leading non-Aboriginal researchers to avoid or tokenize their use. Incorporating sufficient time into funding timelines and agreements to allow for the application of Aboriginal research paradigms, including the involvement of Aboriginal researchers and partners in the analysis and not just the collection of data, is a necessary first step.

## Conclusion

This scoping review has clearly demonstrated that undertaking research with Aboriginal and Torres Strait Islander peoples must be done in conjunction with local communities and this needs to be clearly evident in research publications. Ensuring the involvement of local Aboriginal and Torres Strait Islander communities in all aspects of the research supports the underpinning values of responsibility, reciprocity, respect, spirit and integrity, equity and cultural continuity. It is no longer desirable nor ethically acceptable that any researcher, either Aboriginal and Torres Strait Islander or non-Indigenous, conduct research without community support, engagement, leadership and influence. All future researchers should consider local community involvement in all aspects of the research where the need for the research is generated by the local community and that results, outcomes and benefits of that research are equally shared between the researcher and the communities involved in the research. This needs to involve a review of reportable research guidelines and governance to ensure that researchers respond and report on vital elements of Aboriginal and Torres Strait Islander research. This will ensure that reportable research moves beyond journal publication requirements and supports the underpinning ethical values of Aboriginal and Torres Strait Islander research.

## Biography

Dr Brett Biles is a Murrawarri man from Brewarrina. He has been living on Wiradjuri country for the last 17 years. He holds a Bachelor of Physiotherapy, a Masters in Indigenous Health and a PhD. He is currently the Director of Indigenous Health Education in the Office of Medical Education, UNSW Medicine. Prior to this he was a lecturer in Indigenous Health at the School of Nursing, Midwifery, and Indigenous Health at Charles Sturt University. With a passion for education and health equality, Brett is an early career researcher with a keen interest in Aboriginal men’s health and cardiovascular disease.

Dr Jessica Biles has held an academic position is a Senior Lecturer and at Charles Sturt University since 2008. Her PhD, a phenomenological study that focussed on cultural competence development across a cohort of students within a Bachelor of nursing degree: Australian Indigenous Cultural Competence and Nursing was published in 2017. During my time at CSU, she has held a number of leadership roles and is the co-editor of a text titled Aboriginal and Torres Strait Islander Health and Wellness. Jessica has a strong profile both in teaching and research in the area of cultural competence and equity in healthcare/education. She has experience managing projects and leading teams. Publication and media releases accessible via this link: https://researchoutput.csu.edu.au/en/persons/jbilescsueduau.

Dr Kate Friere has been a physiotherapist since graduating in 1996. She trained in Edinburgh, Scotland and worked in the UK and USA before moving to Australia, with her family, in 2003. Kate has worked in public and private clinical health settings across a wide range of areas, including inpatients, outpatients, rehabilitation and occupational health. In Australia, she has lived in three regional locations: Armidale, Wagga Wagga and Albury. Her work experiences in these locations have led to a keen interest in rural health. Kate started work at Charles Sturt University as a sessional lecturer in health and physiotherapy subjects, combining this with clinical roles in occupational health and public hospital settings. She joined the physiotherapy team in 2019 before moving her position at Three Rivers in January 2020.

Dr Linda Deravin is a proud Wiradjuri woman who has over 30 years’ experience in the nursing profession and has a wide range of nursing expertise having worked in specialities such as aged care, primary healthcare, emergency care, peri-operative care, forensic nursing, leadership and nursing management. Linda has experience in both the public and private sector of the health industry. Currently Linda is the Head of School, Nursing, Paramedicine and Healthcare Sciences, Charles Sturt University. Prior to joining the Charles Sturt team, Linda worked for over 10 years in senior nursing leadership and management roles ranging from Nurse Unit Manager to District Director of Nursing and Midwifery. Linda has a keen interest in Indigenous health and nursing workforce issues and completed a PhD on this topic through Charles Sturt University (CSU). Linda has multiple qualifications in nursing and affirms a strong commitment to nursing education and building a skilled nursing workforce for the future. See publications list at https://researchoutput.csu.edu.au/en/persons/lmalon06csueduau.

Ms Jayne Lawrence is a lecturer in the School of Nursing, Paramedicine and Healthcare Sciences. Jayne was chosen for this position because of her extensive experience in nursing and midwifery in a variety of clinical settings in rural and regional locations. Jayne has been a Registered Nurse for 35 years and a Registered Midwife for 22 years, Jayne completed her Graduate Certificate in Midwifery and then later her Master of Midwifery at Charles Sturt University. Jayne has worked as a clinical midwifery educator, Maternity unit manager and has practiced in all areas of midwifery including antenatal care, birthing suite, postnatal care, special care nursery and antenatal education. She persistently strives to support changes in health pathways to improve the health of current and future generations. Her strengths lie in communication and engagement in a diverse variety of healthcare contexts with clinicians, students and stakeholders. Jayne is passionate about sharing her skills and knowledge with the next generations of nurses and midwives.

Dr Aryati Yashadhana is a Research Associate, at the Centre for Health Equity Training Research & Evaluation (CHETRE) UNSW, and a Visiting Fellow, School of Social Sciences UNSW. Her work focusses on socioeconomic, cultural and political determinants of health, intersectional health equity (gender, race, class) and international development. Her research has largely taken place in collaboration with Aboriginal and Torres Strait Islander peoples in Australia (NSW and Northern Territory) with a focus on analyzing the cultural dimensions of access in the context of eye health and chronic disease. Dr Yashadhana works from a place of understanding and respect for Aboriginal cultural protocols as foundational to research relationships. Internationally, she’s worked in low and middle income settings (Sub-Saharan Africa, South America, South East Asia, Pacific) and been a lead investigator on various projects commissioned by the World Bank Group centred around access to child eye health services (Malawi, Colombia, Pakistan). Dr Yashadhana specializes in qualitative and participatory methodologies, with a particular interest in the application of critical realist and decolonizing theories, the way marginalized peoples and cultures interface with dominant systems and structures and analyzing access to health systems and services.
